# Research on pedestrian detection method based on multispectral intermediate fusion using YOLOv7

**DOI:** 10.1038/s41598-025-88871-y

**Published:** 2025-05-15

**Authors:** Bo Jiang, Jingyu Wang, Guoyin Ren, Mobin Zhi, Zhijie Yu, Yang Zhang, Pengju Ren, Shidong Jia

**Affiliations:** 1 School of Digital and Intelligence Industry, Inner Mongolia University of Science and Technology, BaoTou, 014010 China; 2 Weizhe Changcheng Network Technology Co., Ltd, Zunxin Road, Baotou, 014070 Inner Mongolia Autonomous Region China

**Keywords:** Computer science, Infrared spectroscopy

## Abstract

This study is based on the YOLOv7 object detection framework and conducts comparative experiments on early fusion, halfway fusion, and late fusion for multispectral pedestrian detection tasks. Traditional pedestrian detection tasks typically use image data from a single sensor or modality. However, in the field of multispectral remote sensing, fusing multi-source data is crucial for improving detection performance. This study aims to explore the impact of different fusion strategies on multispectral object detection performance and identify the most suitable fusion approach for multispectral data. Firstly, we implemented early fusion experiments by merging multispectral data with visible light data at the network’s input layer. Next, halfway fusion experiments were conducted, merging multispectral data and visible light data at the network’s middle layers. Finally, late fusion experiments were performed by merging multispectral data and visible light data at the network’s high layers. A comprehensive comparison of the experimental results for various fusion strategies reveals that the halfway fusion strategy exhibits outstanding performance in multispectral pedestrian detection tasks, achieving high detection accuracy and relatively fast speed.

## Introduction

Pedestrian detection is a crucial problem in the field of computer vision, aiming to automatically identify and detect the presence of pedestrians in images or videos. With the growing demand for urban traffic management and security surveillance, pedestrian detection technology has extensive application prospects in intelligent transportation systems, security monitoring, and intelligent robotics. Research on pedestrian detection began in the 1990s, primarily relying on feature-based classification methods, such as edge and contour-based features and Haar features. However, these methods suffer from significant limitations in dynamic and complex environments due to issues like illumination changes, occlusions, and varying pedestrian appearances^1^. In recent years, deep learning methods, particularly Convolutional Neural Networks (CNNs), have revolutionized pedestrian detection by enabling the automatic extraction of features from images, which improves accuracy and robustness. Nonetheless, even with CNN-based methods such as YOLO and Faster R-CNN, problems like low detection accuracy in challenging environments (e.g., poor lighting or occlusions) and high computational costs persist^2^.

To address these challenges, this paper proposes a new approach for multispectral pedestrian detection based on the YOLOv7 framework. Given the complementary nature of RGB and infrared (IR) images, combining these modalities has the potential to enhance detection accuracy, particularly in challenging conditions^3^. Specifically, the goal is to explore various feature fusion strategies—early, halfway, and late fusion—and evaluate their effectiveness in multispectral pedestrian detection. By focusing on the fusion of features from different spectra, this work aims to improve detection robustness and efficiency while reducing computational complexity compared to traditional methods^4^.

Additionally, recent works based on Transformer architectures have demonstrated significant improvements in feature extraction and fusion capabilities, prompting a need to compare such methods against our proposed approach. These Transformer-based methods provide compelling evidence of superior performance, especially in capturing long-range dependencies within images^5^. Therefore, comparing our approach to Transformer-based models can shed light on their respective strengths and weaknesses, particularly with respect to detection accuracy and computational cost^6^.

This study focuses on the task of object detection in multispectral remote sensing images based on the YOLOv7 framework. A series of systematic experiments were conducted to investigate various fusion strategies, including early fusion, halfway fusion, and late fusion, to determine the most suitable approach for multispectral data. This research aims to improve the performance of multispectral remote sensing image object detection while providing critical insights into resource optimization and performance requirements in practical applications^7^.

In the early fusion experiments, multispectral and visible light data were integrated at the input layer of the neural network. This strategy allowed the exploration of low-level feature information from both modalities but exhibited certain deficiencies in capturing high-level semantic information^8^. Previous studies have explored similar fusion methods and identified early fusion as a straightforward, albeit sometimes suboptimal, strategy for object detection in multispectral settings^9^.

Halfway fusion experiments fused multispectral and visible light data at the middle layers of the network, effectively combining semantic information from both modalities and significantly enhancing detection performance^10^. This approach has been widely studied and applied in the context of multispectral pedestrian detection, with notable success in reducing the influence of environmental variables such as illumination changes and occlusions^11^.

Late fusion experiments merged multispectral and visible light data at the high layers of the network. The results indicated that the late fusion strategy achieved higher accuracy in target detection but at the potential expense of detection speed^12^. Researchers have shown that late fusion can be effective in increasing detection precision in complex scenarios, especially when dealing with occlusions or low-contrast regions^13^.

A comprehensive comparison of the experimental results revealed that the halfway fusion strategy outperformed other approaches in multispectral object detection tasks, offering high detection accuracy and relatively fast speeds^14^. This study provides a robust method for target detection in multispectral remote sensing images and holds significant implications for resource optimization and performance requirements in practical applications^15^. Furthermore, advancements in deep learning frameworks, such as the YOLO family, have demonstrated significant progress in real-time pedestrian detection, highlighting the importance of efficient network architectures in such applications^16^.

Future research can further explore different fusion mechanisms and network architectures to enhance the performance and efficiency of multispectral object detection^17^. Such advancements are expected to better meet the demands for high-quality remote sensing data analysis in scientific and engineering fields, providing essential guidance for the development and application of remote sensing image processing^18^.

## Related work

Pedestrian detection methods can be categorized from multiple perspectives. Based on the image source, these methods are classified into visible light detection, thermal imaging detection, and multispectral detection, which combines both visible light and thermal images to leverage their complementary features for improved detection performance^19^. Recent research has shown that multispectral pedestrian detection, which integrates information from both modalities, can significantly enhance detection accuracy, especially in challenging conditions such as low-light environments or adverse weather conditions^20^. These methods have evolved over time to address challenges such as occlusions and variations in pedestrian appearances, with recent advancements utilizing attention mechanisms and cross-modality fusion techniques to further improve detection performance^21^.

### Pedestrian detection based on different image sources

Pedestrian detection is an important research direction in computer vision, with applications in fields such as intelligent transportation and surveillance. Based on the image source, pedestrian detection methods can be categorized into visible light detection, thermal imaging detection, and multispectral detection.

Traditional visible light detection methods rely on features such as image brightness, texture, and shape. With the rise of deep learning, pedestrian detection methods based on Convolutional Neural Networks (CNNs) have significantly improved detection performance. For example, frameworks such as Faster R-CNN and YOLO have achieved excellent results on standard visible light datasets, such as Caltech and City Persons^22^. However, these methods exhibit significant performance degradation in scenarios involving large illumination changes, complex environments (e.g., nighttime or rainy weather), and other challenging conditions, making them unsuitable for real-world applications in such contexts^23^.

Thermal imaging methods capture infrared radiation emitted by objects, offering strong adaptability in low-light and extreme weather conditions, and have gained considerable attention in recent years^24^. Studies have shown that thermal imaging can effectively improve detection capability in occluded scenes. However, due to the low resolution and lack of rich texture information in infrared images, detection performance remains limited in complex backgrounds^25^.

To overcome the limitations of single-modal methods, multispectral pedestrian detection combines the advantages of both visible light and thermal infrared images, leveraging the complementary nature of multimodal information to enhance robustness. The introduction of the KAIST multispectral dataset has provided foundational support for this field^26^. Researchers have explored various feature fusion strategies to improve detection performance. For example, Konig et al. proposed a simple feature concatenation fusion method, but it did not fully exploit the inter-modal relationships^27^. Although multispectral detection methods offer significant advantages in improving detection performance, efficiently fusing multimodal features while balancing inference speed and accuracy remains a key challenge in current research^28^.

### Feature fusion-based pedestrian detection methods

Multimodal feature fusion plays a crucial role in multispectral pedestrian detection. The current feature fusion methods are mainly categorized into early fusion, halfway fusion, and late fusion.

Early Fusion directly concatenates or overlays multimodal data at the input stage, allowing the network to learn inter-modal interaction information in the initial phase. This approach is computationally simple but is easily affected by modal imbalances, making it difficult to capture deep semantic features^29^.

In Halfway Fusion, multimodal features are fused at intermediate network layers using attention mechanisms or convolution operations. This method better balances the detail information of low-level features with the semantic expression of high-level features. A representative method of this type is the Cross Modality Fusion network proposed by Zhang et al. which alleviates the modality mismatch issue by facilitating interaction at specific layers across modalities^30^.

Late Fusion involves weighted fusion of the detection results from each modality at the detection head or output stage. While simple to implement, it fails to fully leverage the feature-level complementary information between modalities^31^.

Recent research trends indicate that halfway fusion strikes a good balance between performance and efficiency, which is why it is widely adopted^32^. However, existing halfway fusion methods do not sufficiently address the differences between modalities, limiting further improvements in detection accuracy and robustness^33^.

### Transformer-based pedestrian detection methods

The success of Transformer architectures has opened new directions for pedestrian detection, with the core self-attention mechanism enabling the capture of global contextual information. This ability offers significant advantages for object detection in complex scenes. In the field of multispectral pedestrian detection, Transformer-based methods have also gained increasing attention^34^.

Kim et al. proposed the MLPD method, which utilizes Transformer models to capture long-range dependencies in multispectral images and improve fusion performance through modality alignment strategies^35^. Zhang et al. further proposed a fusion network based on cross-attention mechanisms, incorporating feature-level modality alignment modules to achieve higher detection accuracy in occluded and complex background scenarios^36^. Shen et al. introduced the ICA-Fusion framework, which improves feature fusion through iterative cross-attention mechanisms, significantly enhancing detection ability in complex backgrounds^37^. Lee et al. also proposed a multimodal pedestrian detection method based on cross-modal reference search, which further enhances detection accuracy through fine-grained reference search across modalities^38^. Despite the accuracy advantages of Transformer-based methods, their high computational cost and slow inference speed limit their applicability in real-time scenarios^39^.

Each of these methods has its advantages and limitations, and researchers choose the appropriate method based on specific application scenarios to enhance the accuracy and robustness of pedestrian detection. For instance, traditional visible light detection methods excel under favorable lighting conditions but struggle in low-light or complex environments, where thermal imaging demonstrates superior adaptability. However, its limited resolution and lack of texture information pose challenges in intricate scenarios, driving the development of multispectral detection techniques. Recent approaches, such as those employing advanced feature fusion strategies like iterative cross-attention and cross-modality reference search, have effectively leveraged the complementary strengths of visible and thermal imaging to achieve improved detection results.

Building on these advancements, this study proposes a novel approach based on the YOLOv7 framework, conducting comparative experiments on early, halfway, and late fusion strategies for multispectral pedestrian detection. The halfway fusion strategy, which merges multispectral and visible light data at intermediate network layers, was found to deliver the best performance, offering a robust balance of high detection accuracy and computational efficiency. This method not only addresses the challenges of multispectral feature integration but also surpasses many existing methods in terms of practical applicability, particularly in complex detection scenarios.

## Proposed method

### Multispectral feature fusion strategy

This network employs YOLOv7 as the foundational framework to achieve rapid and accurate multispectral pedestrian detection. YOLOv7, a deep convolutional neural network, is renowned for its exceptional object detection performance. The network architecture is based on the YOLO series models, yet it incorporates significant innovations and improvements in its design. This model utilizes a series of strategies to enhance the accuracy and efficiency of object detection.

The architecture of YOLOv7 is designed with a deeply scalable and modular approach, ingeniously integrating convolutional layers and feature extraction modules of various scales. This design philosophy enables the network to perceive and predict objects at multiple scales, significantly enhancing the robustness and accuracy of detection. In addition to the architectural innovations, YOLOv7 incorporates attention mechanisms and multi-scale fusion strategies. These techniques allow the network to better focus on critical features, thereby further improving detection precision. During the training and optimization phases, YOLOv7 employs a series of advanced techniques. These include improved data augmentation methods, optimized loss functions, and efficient training strategies. The combination of these techniques enables YOLOv7 to adeptly handle object detection tasks across various scenarios and scales. Moreover, YOLOv7 achieves optimized inference speed while maintaining high accuracy. This is primarily attributed to its optimized network structure and computational efficiency. Consequently, YOLOv7 is an ideal choice for real-time object detection and large-scale data processing tasks.

**Fig. 1:**
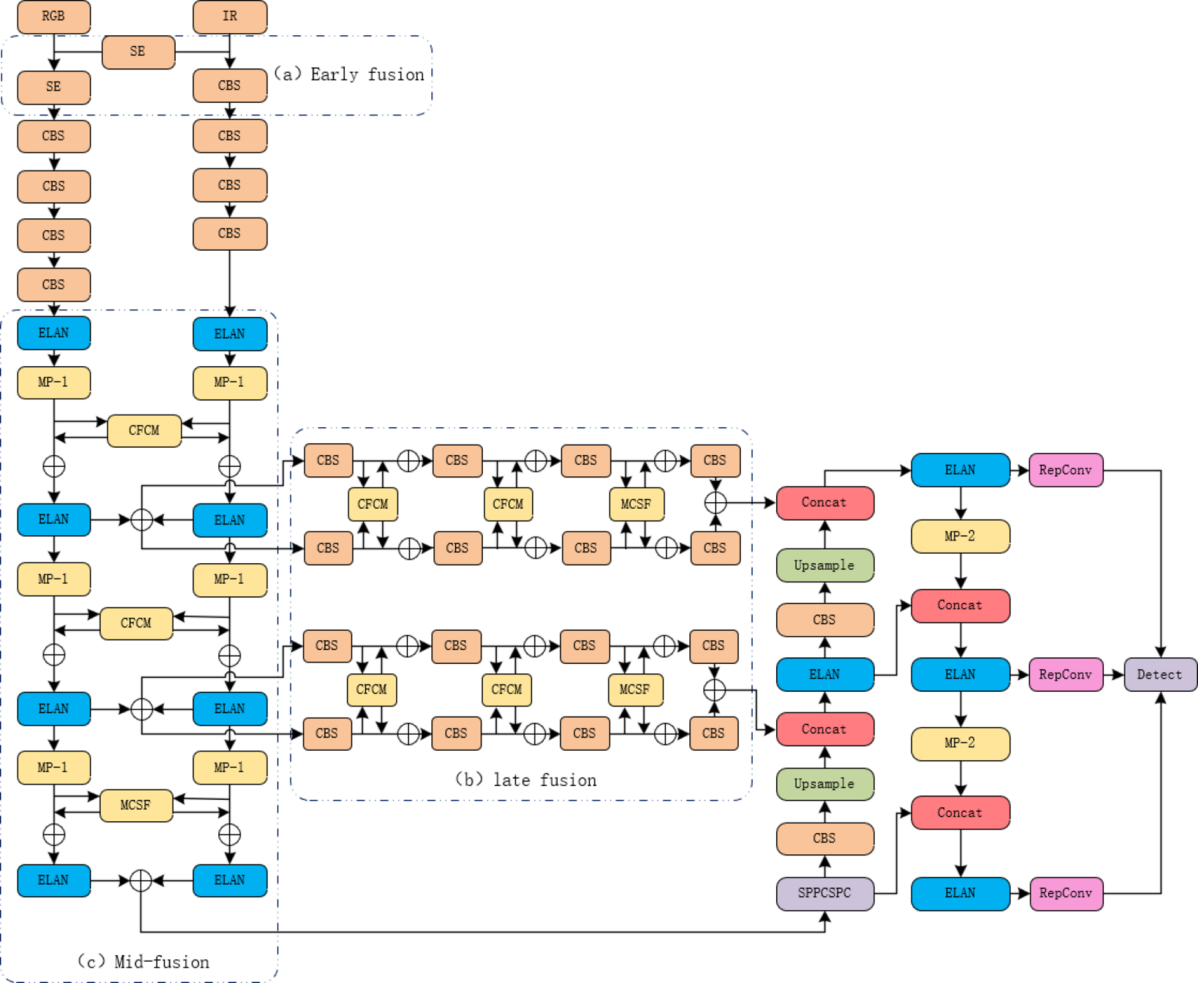
Yolov7 improvement network diagram.

This study specifically addresses the issue of multimodal data fusion, particularly when RGB images and thermal images are used as inputs. To fully exploit the information from both modalities, we constructed a cross-modal fusion network based on YOLOv7. This network comprises a shallow sub-network and a deep sub-network. The shallow sub-network is responsible for extracting simple geometric features, while the deep sub-network generates rich semantic information. By appropriately fusing features from different layers, the network achieves superior multimodal data fusion performance. RGB images and thermal images are separately input into the two sub-networks, which handle feature extraction and fusion accordingly. This approach allows us to fully utilize the information from both modalities, further enhancing the accuracy and robustness of object detection. Figure 1 illustrates the YOLOv7 network architecture.

The fusion framework simultaneously employs the MCSF (Multi-Scale Cross-Spectrum Fusion) module and the CFCM module (Cross-Modal Feature Complementary Module) to achieve superior detection performance. Since all modules are integrated into the network and trained end-to-end, the loss function is defined as follows:

1 $$\begin{array}{c}Loss={L}_{local}+{L}_{conf}+{L}_{cls} \end{array}$$

**Fig. 2:**
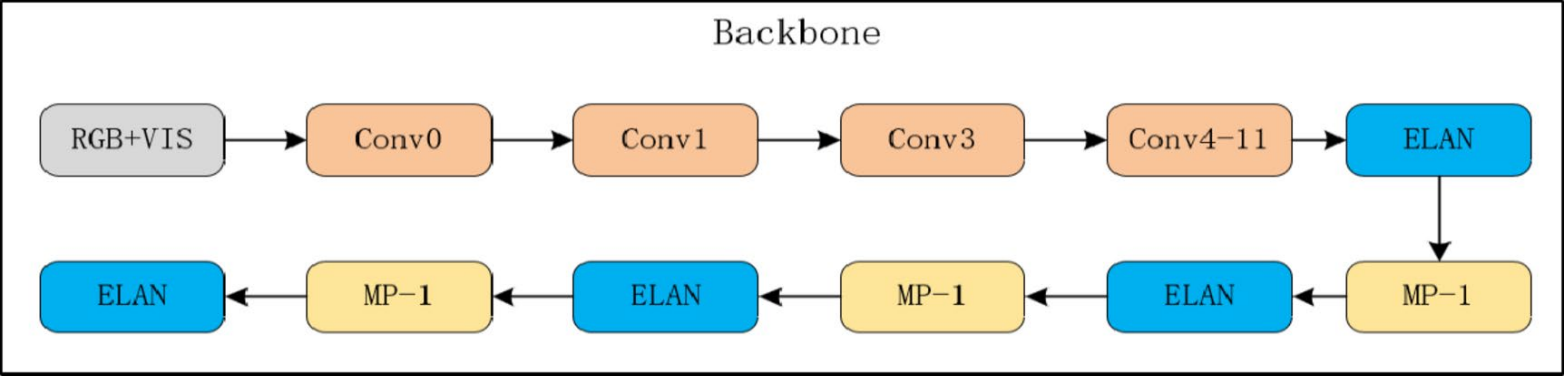
Yolov7 early fusion.

Early fusion, mid-fusion, and late fusion are commonly used feature fusion strategies in the field of multispectral image processing and object detection. These strategies are employed to merge image information from different bands or modalities to enhance target detection performance. Below is a brief introduction to these fusion strategies:

Early fusion is a strategy where multispectral data and visible light data are combined at the input layer of the neural network. This means that multispectral and visible light images are merged at the pixel level into a single input before processing. This approach allows the neural network to simultaneously process information from multiple bands, including low-level features. Early fusion is typically capable of capturing low-level feature information from both multispectral and visible light data, but it may have certain limitations in capturing high-level semantic information. Figure 2 illustrates the integration of early fusion into the YOLOv7 backbone.

**Fig. 3:**
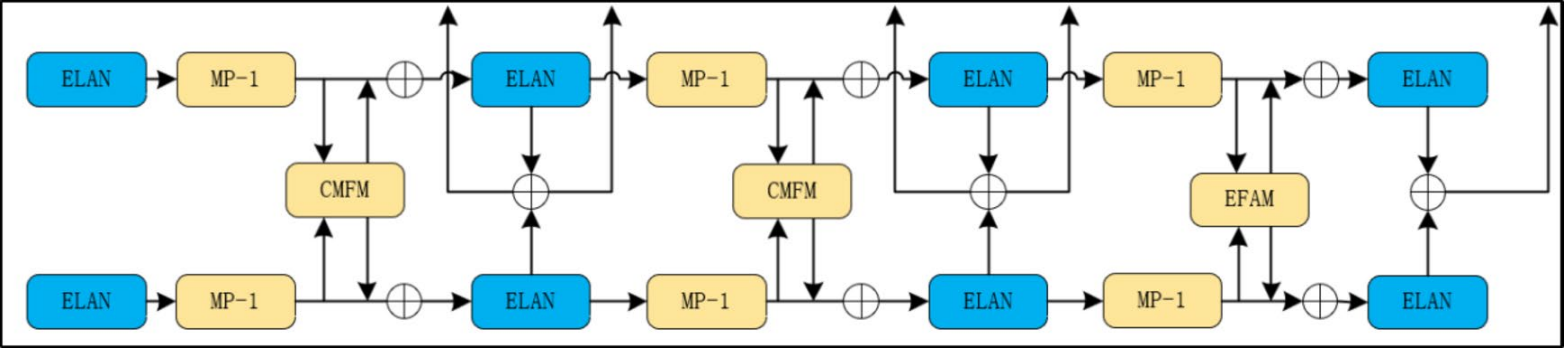
Yolov7 mid-fusion.

The mid-fusion strategy merges multispectral data and visible light data at the intermediate layers of the neural network, typically within the convolutional or pooling layers. This method allows the multispectral and visible light data to interact at a certain depth within the network, thereby better combining their semantic information. Mid-fusion often improves detection performance by capturing higher-level information within the neural network. Figure 3 illustrates the integration of mid-fusion into the YOLOv7 backbone.

**Fig. 4:**
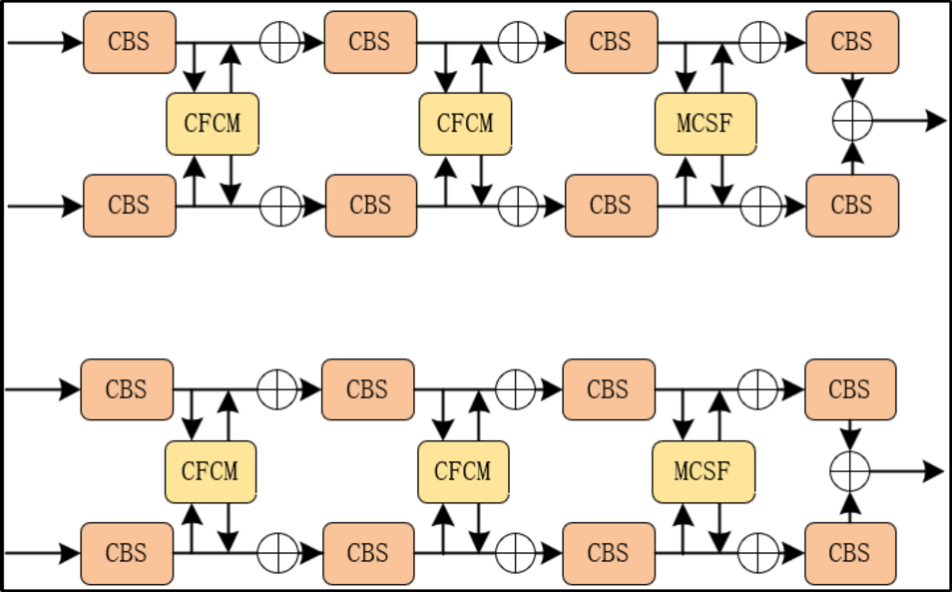
Yolov7 late fusion.

Late fusion is a strategy that merges multispectral data and visible light data at the higher layers of the neural network, usually after feature extraction. This method can achieve higher accuracy in object detection because it allows the features from each modality to be fused after being independently extracted, thereby fully leveraging the advantages of each modality. However, late fusion might sacrifice some detection speed. Figure 4 illustrates the integration of late fusion into the YOLOv7 backbone.

### Multi-spectral feature fusion module

#### CFCM module

Under good lighting conditions, RGB images provide detailed information and contour details. On the other hand, thermal images are less affected by lighting conditions and provide clear pedestrian contour information. To enable the detection network to achieve all-weather pedestrian detection, the CFCM module facilitates the interaction of features from both modalities. By utilizing complementary information provided by the other modality, the CFCM module can partially recover objects lost in one modality, thereby reducing object loss and conveying more information.

The CFCM module operates by converting features from different modalities into the other modality during feature extraction, thereby obtaining complementary information from the other modality. This allows both modalities to learn more complementary features. The working principle of the CFCM module is as follows: First, the channel differential weighting method is used to obtain the differential features of the two modality feature maps. Second, different features from different modalities are amplified and fused with features from the other modality. Finally, to enable the network to focus on important features, channel attention operations are performed on the feature maps of both modalities, and the features are fused. According to previous studies, the specific workflow of the CFCM module is as follows:

2 $$\begin{array}{c}{F}_{D}={F}_{R}-{F}_{I} \end{array}$$

Which, ${F}_{R}\in {\mathbb{R}}^{C\times H\times W}$ represents the RGB convolutional feature map, while ${F}_{T}\in {\mathbb{R}}^{C\times H\times W}$ represents the thermal convolutional feature map. ${F}_{D}\in {\mathbb{R}}^{C\times H\times W}$ is obtained through channel-wise differential weighting of these two features.

3 $$\begin{array}{c}{F}_{TD}=\sigma \left(GAP\left({F}_{D}\right)\right) \odot {F}_{T} \end{array}$$

4 $$\begin{array}{c}{F}_{RD}=\sigma \left(GAP\left({F}_{D}\right)\right) \odot {F}_{R} \end{array}$$

Which, GAP denotes global average pooling, σ represents the sigmoid activation function, and $\odot$ denotes element-wise multiplication. ${F}_{TD}\in {\mathbb{R}}^{C\times H\times W}$ and ${F}_{RD}\in {\mathbb{R}}^{C\times H\times W}$ are obtained through feature enhancement, suppression, and fusion with ${F}_{R}$.

5 $$\begin{array}{c}{{F}^{{\prime }}}_{T}=\mathcal{F}\left({F}_{T}\parallel {F}_{RD}\right)\oplus \sigma \left(GAP\left({F}_{T}\right)\right) \end{array}$$

6 $$\begin{array}{c}{{F}^{{\prime }}}_{R}=\mathcal{F}\left({F}_{T}\parallel {F}_{TD}\right)\oplus \sigma \left(GAP\left({F}_{R}\right)\right) \end{array}$$

**Fig. 5:**
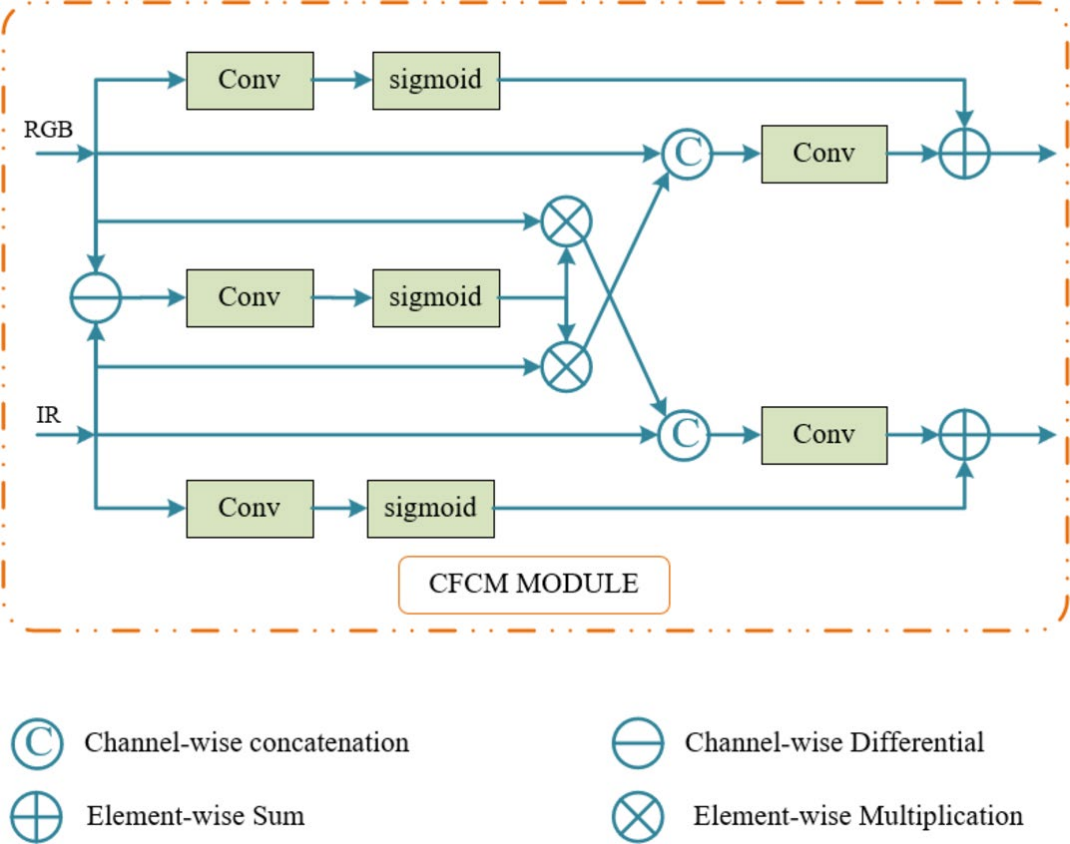
CFCM Module.

Which, $\left|\right|$ represents channel-wise concatenation operation, $\oplus$ denotes element-wise addition, and$\mathcal{F}\left( \right)$ represents the residual function. ${{F}^{{\prime }}}_{T}\in {\mathbb{R}}^{C\times H\times W}$ is the fusion of $\mathcal{F}\left({F}_{T}\parallel {F}_{RD}\right)\in {\mathbb{R}}^{2C\times H\times W}$ and ${F}_{T}$ after feature enhancement. ${{F}^{{\prime }}}_{R}\in {\mathbb{R}}^{C\times H\times W}$ is the fusion of $\mathcal{F}\left({F}_{R}\parallel {F}_{TD}\right)\in {\mathbb{R}}^{2C\times H\times W}$ and ${F}_{R}$ after feature enhancement. Through these operations, the complementary and fused information from both modalities is obtained. This enriched feature map is then sent to the network for further feature extraction. Figure 5 illustrates the structure of the CFCM module.

#### MCSF module

In multispectral pedestrian detection, the fusion of data from different modalities is a crucial step. An effective fusion method should be able to supervise the integration of information from various modalities to enhance the detector’s performance. Existing multispectral feature fusion methods mainly include the SUM and MIN methods. The SUM operator represents the element-wise summation of features, which can be considered a linear feature fusion with equal weights. The MIN method involves performing a 1 × 1 convolution to reduce the dimensionality of the concatenated multimodal features; this is an unsupervised nonlinear feature fusion. Therefore, it is reasonable to design a feature fusion method that uses illumination conditions as a supervisory condition.

**Fig. 6:**
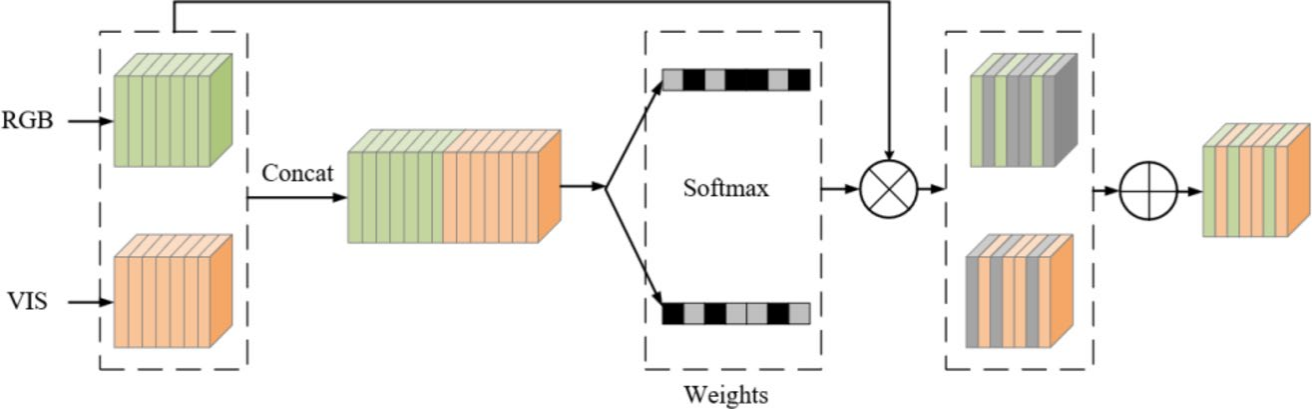
MCSF module.

Under different lighting conditions, the roles of color images and thermal images vary. Apart from specific cases (such as standing in the shadows), most pedestrians are well-illuminated during the day; however, thermal images are not sensitive to lighting. Conversely, thermal imaging can better capture the visual characteristics of pedestrians at night. Therefore, it is logical to design a supervised feature fusion method using illumination. As shown in Fig. 6, we introduce the MCSF module, which can adaptively adjust the channel features between color and thermal modalities to achieve optimal fusion under different lighting conditions. Implementing the MCSF requires three steps. In the first stage, the MCSF concatenates features from the color and thermal modalities along the channel dimension. Then, in the second stage, it uses learned weights to adaptively aggregate these features.

In the first step, features from the color (C) and thermal (T) branches are connected in the channel dimension. The connected feature mapping can be represented as.

7 $$\begin{array}{c}{F}_{i}=Concat({C}_{i},{T}_{i}) \end{array}$$

where *C*_*i*_ and *T*_*i*_ are defined as the color and thermal characteristics of the i-th layer. ${C}_{i},{T}_{i}\in {\mathbb{R}}^{\frac{H}{r}\times \frac{W}{r}\times C}$

In the second step, the channel feature vector F1 is generated using Global Average Pooling (GAP) and Global Maximum Pooling (GMP). The formula for the c-th channel element in GAP and GMP is as follows.

8 $$\begin{array}{c}{F}_{C}^{1}=GAP\left({X}_{C}\right)+GMP\left({X}_{C}\right) \end{array}$$

Then, a new compact feature *F*^2^ is created that adaptively learns the fusion weights of the color and thermal features. This is achieved by having a fully connected (FC) layer with lower dimensionality.

9 $$\begin{array}{c}Z={F}^{2}=FC\left({F}^{1}\right) \end{array}$$

which ${F}^{1}\in {\mathbb{R}}^{C},{F}^{2}\in {\mathbb{R}}^{{C}^{{\prime }}},{C}^{{\prime }}=max\left(C/r,L\right)$ is a typical setup in our experiments.

In addition, softmax is used for normalization and the learned weights ${\alpha }_{c}$ and ${\beta }_{c}$ are used to select the corresponding level features for the final fusion Fc. Note that ${\alpha }_{c}$ and ${\beta }_{c}$ are just the scalar values of channel c and ${\alpha }_{c}{,\beta }_{c}\in \left[\text{0,1}\right]$.

10 $$\begin{array}{c}{\alpha }_{c}=\frac{{e}^{{A}_{c}z}}{{e}^{{A}_{c}z}+{e}^{{B}_{c}z}} \end{array}$$

11 $$\begin{array}{c}{F}_{c}{=\alpha }_{c}\cdot {C}_{c}^{i}+{\beta }_{c}\cdot {T}_{c}^{i} \end{array}$$

Here, ${F}_{c}\in {\mathbb{R}}^{\frac{H}{r}\times \frac{W}{r}},{\alpha }_{c}+{\beta }_{c}=1,A,B\in {\mathbb{R}}^{{C\times C}^{{\prime }}}$. This method adaptively aggregates features at all levels for each scale. The output of MCSF can serve as input to a new fusion branch for feature extraction.

## Experimental results

This section introduces the datasets, evaluation metrics, and implementation details used in the experiments. The proposed method is evaluated using the KAIST and UTOKYO multispectral pedestrian detection datasets, leveraging metrics such as precision, recall, F_1_ score, mAP (mean Average Precision), and FPS to ensure a comprehensive assessment of detection performance. YOLOv7 was selected as the baseline for comparison due to its balance between high efficiency and accuracy, making it a benchmark in real-time object detection. Additionally, comparisons were made with other state-of-the-art methods, including both single-stage and two-stage detection algorithms, to establish the performance improvements in terms of accuracy, computational cost, and inference speed.

The choice of YOLOv7 as the baseline highlights its relevance in tasks requiring real-time performance. While two-stage methods like Faster R-CNN demonstrate high precision, they struggle with inference speed, making them less suitable for real-time applications. In contrast, single-stage methods like SSD and earlier YOLO versions offer better speed but compromise on precision. Multispectral approaches such as Fusion RPN + BF and CIAN demonstrate strong capabilities in complex environments but face challenges in real-time deployment due to computational overheads. By addressing these limitations, the proposed method ensures both enhanced accuracy and efficiency through innovative feature fusion strategies and model optimizations.

Finally, ablation experiments are conducted to analyze the contributions of two critical modules and variations in the model architecture. These experiments provide insights into how each component improves overall detection performance, further validating the robustness and practicality of the proposed approach.

### Datasets

The KAIST Multispectral Pedestrian Dataset is evaluated as a commonly used multispectral pedestrian dataset. The KAIST dataset was collected using a visible light camera and an infrared thermal imager, resulting in 95,328 pairs of color-thermal images, with 50,172 pairs for the training set and 45,156 pairs for the test set. Following the sampling principles described in^7,12^, every second frame of the training videos was sampled, removing instances with heavy occlusion and small individuals (< 50 pixels). The resulting training set contains 7,601 color-thermal image pairs. The test set was sampled every 20 frames, resulting in 2,252 color-thermal image pairs. Annotations for the training and test sets of the KAIST dataset were improved. The dataset includes pedestrians under various lighting conditions, scales, and levels of occlusion, making them challenging to detect.

The UTOKYO Dataset is a multispectral dataset captured during both daytime and nighttime using four different cameras (RGB, FIR, MIR, and NIR) mounted on an autonomous vehicle. A total of 7,512 sets of images were captured, with 3,740 sets during the day and 3,722 sets at night. Pedestrians were annotated in this dataset. It uses 1,466 sets of correctly aligned images, each sized 320 × 256 pixels, as the test set.

To evaluate our method, the log-average miss rate (MR) over false positives per image (FPPI) in the range $[{10}^{-2},{10}^{0}]$(denoted as ${MR}_{-2}$) is used to measure pedestrian detection performance. Experiments were conducted under reasonable settings, generally defined as pedestrians with heights greater than 55 pixels, with all settings having heights greater than 20 pixels.

### Evaluation

In this study, the evaluation metric used is the log-average miss rate (MR), which is widely applied in pedestrian detection. Specifically, the detection bounding boxes $\left({BB}_{d}\right)$ generated by the model are compared with the ground truth bounding boxes $\left({BB}_{g}\right)$. An Intersection over Union ($\text{I}\text{O}\text{U}$) greater than a threshold indicates a match between$\left({BB}_{d}\right)$ and $\left({BB}_{g}\right)$. The $\text{I}\text{O}\text{U}$ is defined as follows:

12 $$\begin{array}{c}IoU=\frac{area\left({BB}_{d}\cap {BB}_{g}\right)}{area\left({BB}_{d}\cup {BB}_{g}\right)} \end{array}$$

Detection bounding boxes $\left({BB}_{d}\right)$ that do not match are marked as false positives (FP), ground truth bounding boxes $\left({BB}_{g}\right)$ that do not match are marked as false negatives (FN), and matched $\left({BB}_{d}\right)$ and $\left({BB}_{g}\right)$ are marked as true positives (TP). The miss rate (MR) is defined as the ratio of the total number of missed detections to all positive samples:

13 $$\begin{array}{c}Miss\; Rate==\frac{FN}{FN+TP} \end{array}$$

Let $Num\left(img\right)$ be the number of images in the test set. Then the False positive Per Image (FPPI) is expressed by the following equation:

14 $$\begin{array}{c}FPPI==\frac{FP}{Num\left(img\right)} \end{array}$$

From the equation, the smaller the MR of the algorithm, the better the network detection performance. To further evaluate the model, additional metrics are introduced: Precision, Recall, ${F}_{1}$ Score. Precision is the ratio of correctly predicted positive samples to the total number of positive predictions.

15 $$\begin{array}{c}Precision==\frac{TP}{TP+FP} \end{array}$$

Recall is the ratio of correctly predicted positive samples to the total number of ground truth positive samples.

16 $$\begin{array}{c}Recall==\frac{TP}{TP+FN} \end{array}$$

${F}_{1}$ Score is the harmonic means of precision and recall, balancing both metrics.

17 $$\begin{array}{c}{F}_{1}==2\cdot \frac{Precision\cdot Recall}{Precision+Recall} \end{array}$$

## Experiment

Our method is implemented using the same configuration as YOLOv7. For training with the KAIST and UTOKYO datasets, the input image size is set to 640 × 640 pixels. If a label in the ground truth is “person” and the height is greater than 50 pixels, it is included in the training set; otherwise, it is marked as ignored. In the UTOKYO experiments, only RGB and FIR images are used as input images for comparison. Before training, anchor points are obtained using the k-means clustering method. In the KAIST experiments, the anchors are (16,38), (22,53), (31,74), (43,102), (59,141), (82,196), (113,271), (156,375), and (216,520). In the UTOKYO experiments, the anchors are (13,24), (18,33), (24,45), (32,76), (44,106), (82,196), (154,206), (206,324), and (293,478).

During the testing phase, the original size of the input images is used to predict height, offset, and position. We first select bounding boxes with scores higher than 0.001, then apply Non-Maximum Suppression (NMS) with an overlap threshold of 0.65 for final processing.

Specifically, our multispectral pedestrian detection method is trained using the Stochastic Gradient Descent (SGD) optimizer, with an initial learning rate of 0.0001 and a learning rate schedule set for steps. The number of training epochs is set to 100, and each batch is constructed from 8 images. The experiments are conducted using Python (version 3.7) and PyTorch (version 1.10.0) for ASP model training, experimental platform runs on Ubuntu 20.04, with a Intel(R) Core (TM) i7-10700 CPU and an RTX 3090Ti GPU (24GB).

**Fig. 7:**
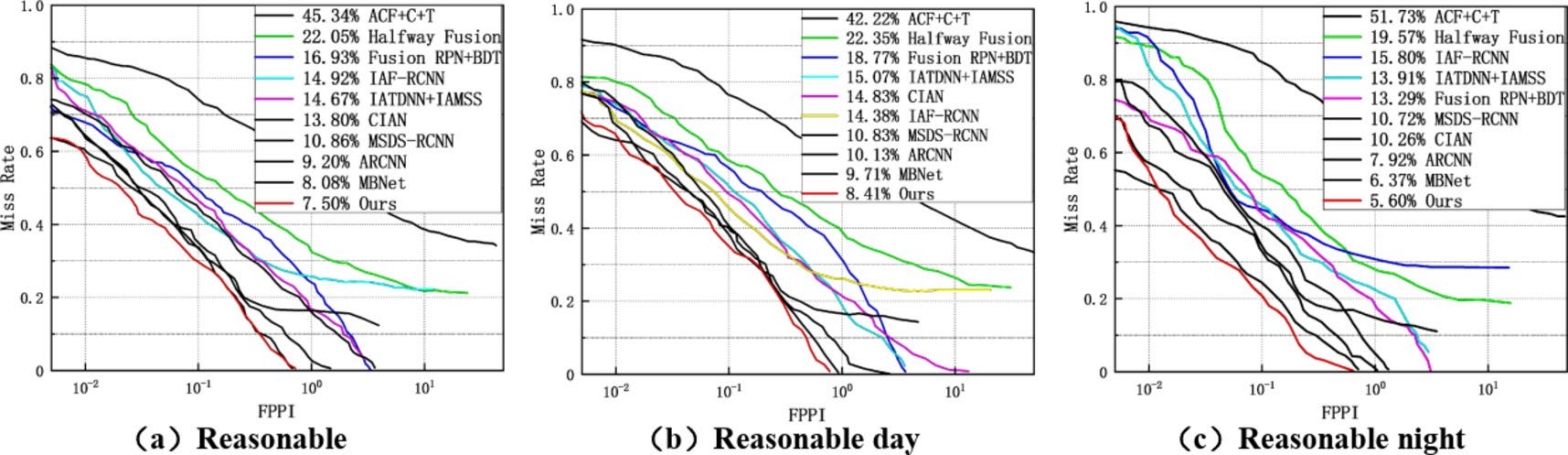
Under reasonable settings (**a**) all; (**b**) daytime; (**c**) nighttime..

The proposed fusion method is evaluated under reasonable and all settings by comparing it with ACF + T + THOG, Fusion, Fusion RPN + BDT, IAF R-CNN, IATDNN + IASS, CIAN, ms-rcnn, ARCNN, MBNet, and Fusion CSP Net. Among these detection methods, Fusion CSP Net and our method are one-stage methods, while the others are two-stage methods. The experimental results in Fig. 7a show that our detection method outperforms all these methods, with the lowest MR of 7.50% under reasonable settings. In Fig. 7b,c, it can be seen that our method also achieves excellent performance under reasonable settings for both day and night. However, our method performs better at night than during the day, indicating that our proposed detection method is more suitable for pedestrian detection under dark lighting conditions.

**Fig. 8:**
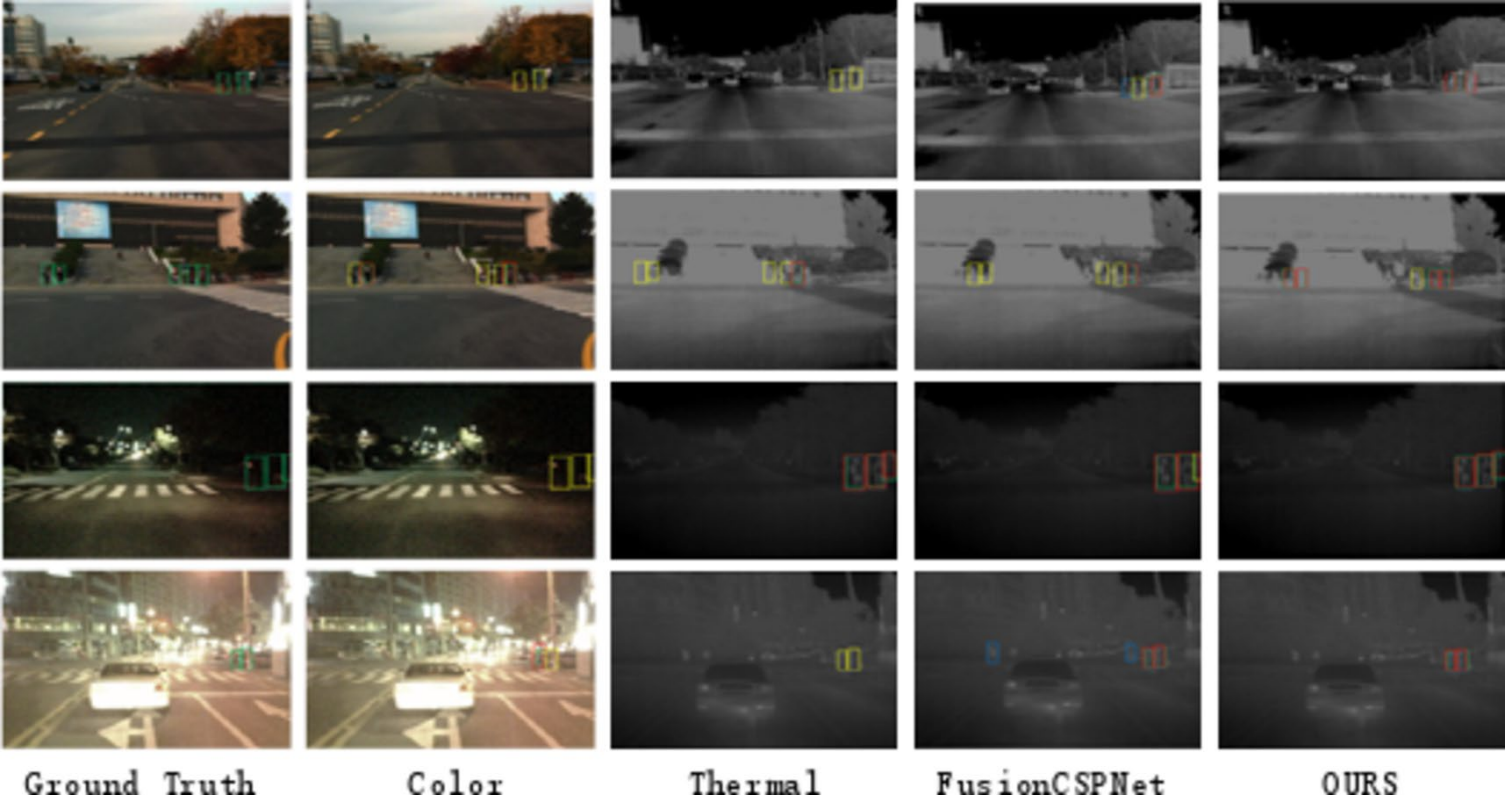
Qualitative comparison of multispectral pedestrian detection results with other state-of-the-art methods in the KAIST rational test subset.

To provide a more intuitive comparison of the detection results from these detectors, we conducted a qualitative evaluation of four multispectral pedestrian detectors on the Reasonable test subset, as shown in Fig. 8. Similar trends were observed in other reasonable daytime and nighttime subsets.

**Fig. 9:**
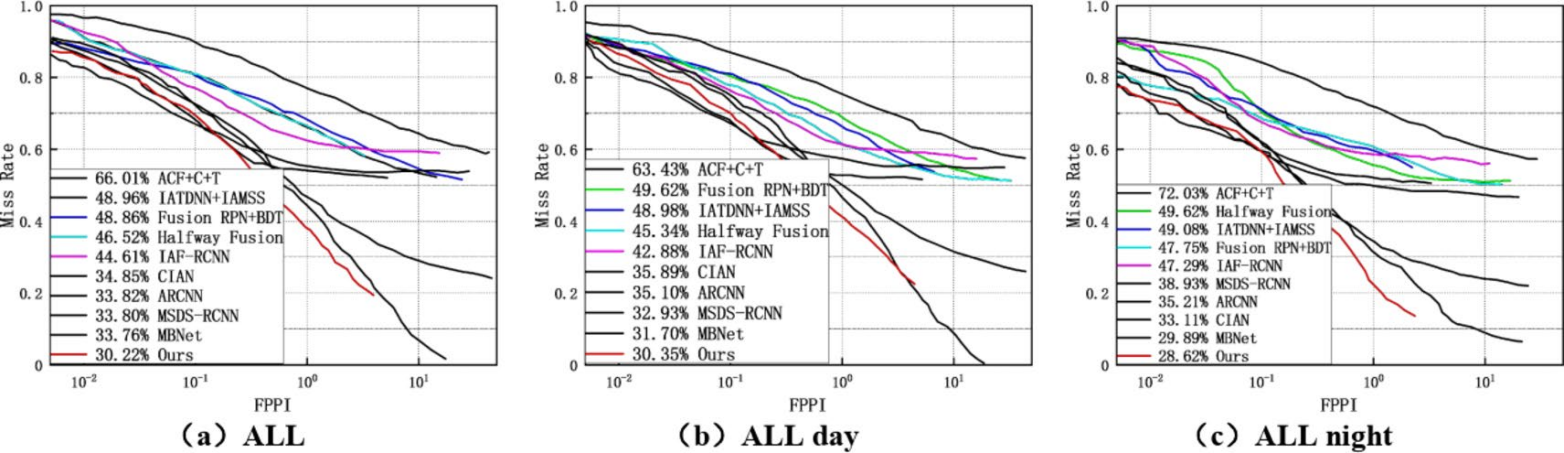
Under the All setting (**a**) All; (**b**) Day; (**c**) Night.

We also found that, compared to state-of-the-art methods, our approach achieved the best accuracy on the KAIST dataset across all settings, as shown in Fig. 9a–c. This performance improvement highlights the robustness of our method in handling varying conditions. Specifically, our approach demonstrated superior ability to detect and accurately classify individuals across different scales, ranging from close-up to far-off subjects. The results suggest that our method is highly effective in distinguishing people at various scales, even in challenging scenarios, making it particularly suitable for real-world applications such as surveillance and security systems.

**Fig. 10:**
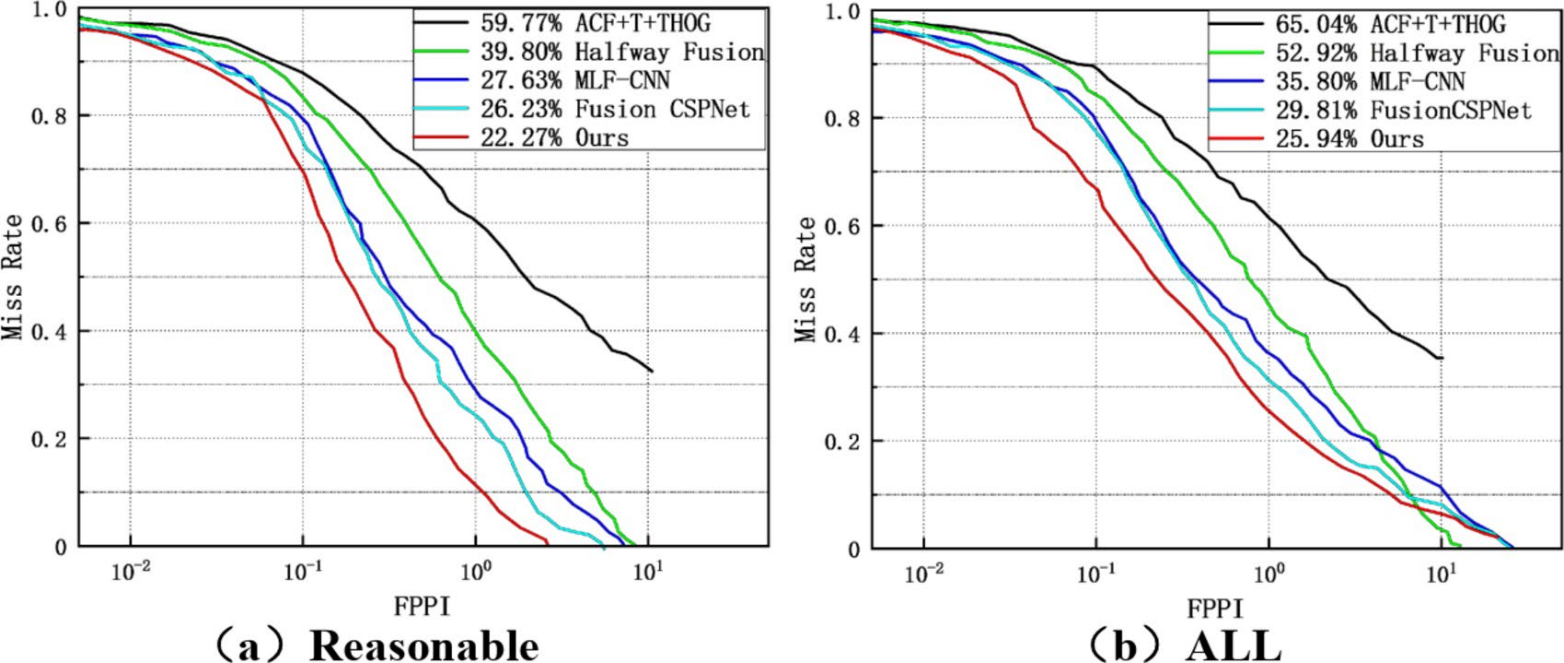
MR under (**a**) reasonable setting; (**b**) all settings.

As shown in Fig. 10, we also evaluated the proposed method on the UTOKYO test dataset, comparing it under both reasonable and all settings with ACF + T + THOG, Halfway Fusion, MLF-CNN, and Fusion-CSPNet. ACF + T + THOG, Halfway Fusion, and MLF-CNN are two-stage detection methods, while Fusion-CSPNet and our method are single-stage methods. Among the existing detectors, Fusion-CSPNet performs the best with a miss rate (MR) of 26.23% under reasonable settings and an MR of 29.81% under all settings. Using our method, we achieved MR values of 22.27% and 25.94%, respectively, clearly demonstrating that our method achieved the best performance.

**Fig. 11:**
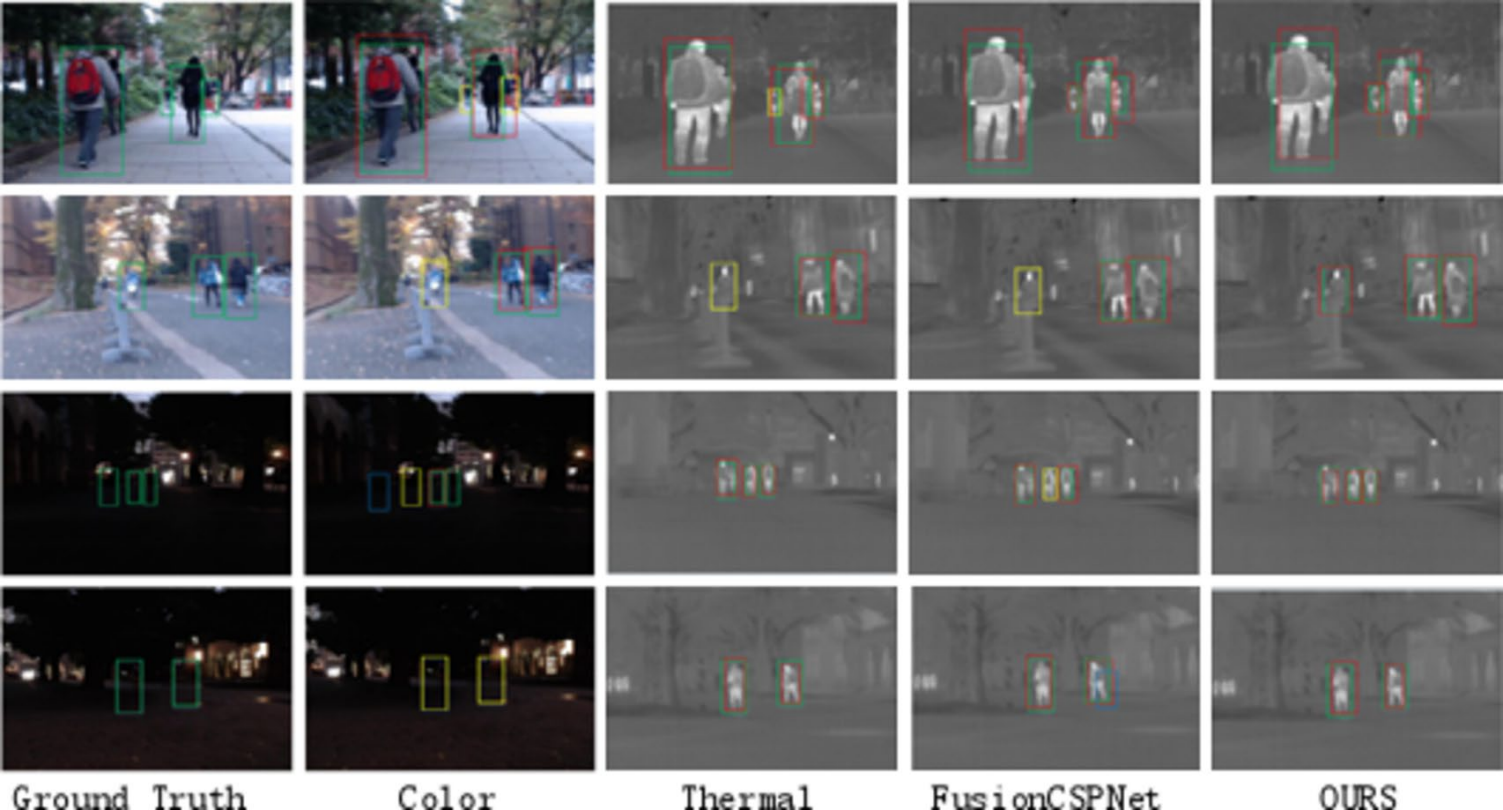
Qualitative comparison of multispectral pedestrian detection results with other state-of-the-art methods in the KAIST rational test subset.

As shown in Figs. 7 and 9, and 10, our method achieved significant performance compared to other methods, especially two-stage methods. This demonstrates that our single-stage detection approach is more suitable for multispectral pedestrian detection. Furthermore, in Fig. 11, we qualitatively showcase some sample detection results.

Table 1 illustrates the computational cost and performance of our method compared to state-of-the-art methods. The proposed multispectral detection method achieves a runtime of only 0.08 s per image (equivalent to 12.5 FPS) on a single NVIDIA Tesla P40 GPU, highlighting its computational efficiency. Additionally, the proposed method demonstrates superior detection performance, achieving a precision of 83.5%, a recall of 82.3%, an F1-score of 82.9%, a mean Average Precision (mAP) of 85.7% and a MR of 7.50%. These results indicate that our single-stage multispectral detection method not only outperforms two-stage methods in terms of runtime efficiency but also surpasses other methods in detection accuracy and robustness. This makes it highly suitable for real-time applications in challenging environments.

**Table 1:** Comparison with other multispectral detectors on the KAIST dataset.

Method	Runtime (s)	Precision (%)	Recall (%)	F1-score (%)	mAP (%)	MR (%)
ACF	2.73	65.0	62.8	63.9	70.0	27.5
Halfway fusion	0.43	70.5	68.9	69.7	74.0	23.0
Fusion RPN + BF	0.8	74.8	72.5	73.6	78.5	18.0
IAF R-CNN	0.21	78.2	76.9	77.6	82.0	16.0
IATDNN + IASS	0.25	79.5	78.0	78.7	83.3	14.5
CIAN	0.07	81.0	80.0	80.5	84.5	12.0
MSDS-RCNN	0.22	81.8	81.0	81.4	85.0	10.5
ARCNN	0.12	80.8	79.5	80.1	84.0	13.5
MLPD	0.12	–	–	–	–	7.58
GAFF	0.09	–	–	–	72.9	10.62
ICA-fusion	–	–	–	–	79.2	7.17
OURS	0.08	83.5	82.3	82.9	85.7	7.5

### Ablation experiments

#### Multispectral data enhancement

In this experiment, the impact of each augmentation technique was isolated by adding only the specific augmentations listed to the baseline enhancement of the fusion model. To provide a detailed comparison of the augmentations presented in Table 2, we can make several observations. Firstly, the addition of simple noise augmentation alone did not result in a significant improvement in performance. However, when we sampled the individual probability of noise augmentations, represented by the term “asynchronous,” the performance of noise augmentation showed the best results. Although these improvements were minor, this finding suggests that employing different noise augmentations for different modalities could be advantageous.

Furthermore, both random masking and synchronous random erasing consistently enhanced our baseline model. These techniques independently contributed to performance gains. Notably, the combination of random masking and random erasing led to a further reduction in the miss rate across both subsets examined in the study. This indicates that while each augmentation technique on its own can provide benefits, their combined application amplifies the overall enhancement effect. This comprehensive analysis underscores the importance of considering multiple augmentation strategies to optimize the performance of the fusion model.

**Table 2:** Impact of multispectral data enhancement.

Augmentation	Reasonable [%]		ALL [%]	
	Average MR	Min. MR	Average MR	Min. MR
Baseline augmentation	7.40 ± 0.26	7.13	23.96 ± 0.24	23.68
+Gaussian on VIS	8.07 ± 0.63	7.62	24.33 ± 0.79	23.76
+Gaussian on VIS, Gaussian on IR, Async.	7.78 ± 0.20	7.56	23.90 ± 0.71	23.27
+Gaussian on VIS Poisson on IR, Async.	7.77 ± 0.64	7.25	23.95 ± 0.90	22.92
+Gaussian on VIS Salt and Pepper on IR, Async.	7.52 ± 0.26	7.27	23.82 ± 0.25	23.65
+Random Erasing, Async.	7.52 + 0.22	7.31	24.08 ± 0.84	23.47
+Random Erasing, Sync.	7.29 ± 0.33	7.03	23.58 ± 0.54	23.1
+Random Masking	7.02 ± 0.59	6.44	23.0 ± 0.21	22.85
+Random Masking + Random Erasing, Async.	6.84 ± 0.14	6.69	22.66 ± 0.11	22.54
+Random Masking + Random Erasing, Sync.	6.96 ± 0.48	6.42	22.29 ± 0.33	21.95

A comparison of different data augmentations is provided. “Asynchronous” means that augmentations are applied independently to the two modalities, while “synchronous” means that augmentations are applied synchronously to both modalities.

#### Multispectral fusion architecture

To evaluate our fusion architecture, we also implemented comparisons with color-only and thermal-only detection. As shown in Table 3, the MR values for pure color detection and pure thermal detection on the KAIST dataset under reasonable settings are 20.50% and 16.64%, respectively, while for the UTOKYO dataset, the MR values are 33.55% and 32.18%, respectively. It is evident that for KAIST, thermal-only detection performs better than color-only detection by several points, indicating that the thermal sensor is more useful in multispectral pedestrian datasets. Among these multimodal feature fusion architectures, input fusion performs the worst under both reasonable and all settings. The gap between single-modal and multimodal methods is substantial. For KAIST, early fusion achieves an MR of 6.06%. For UTOKYO, the MR for early fusion is 28.80%. This demonstrates that multimodal pedestrian detection can significantly enhance detection performance.

**Table 3:** Impact of multispectral fusion architecture.

Method	KAIST		UTOKYO	
	Reasonable (%)	ALL (%)	Reasonable (%)	ALL (%)
Color only	20.50	43.06	33.55	36.74
Thermal only	16.64	35.32	32.18	35.32
Input fusion	6.06	23.15	28.80	32.62
Early fusion I	5.99	22.50	24.21	27.51
Early fusion II	5.65	22.25	24.91	28.82
Halfway fusion	4.91	20.64	23.14	26.21
Late fusion	5.35	22.07	24.86	27.93

Among these multimodal feature fusion architectures, halfway fusion outperforms other fusion architectures under both reasonable and all settings. For the KAIST and UTOKYO datasets, the MR values under reasonable settings for halfway fusion are 4.91% and 23.14%, respectively. The difference in MR values between “early fusion” and “halfway fusion” for KAIST and UTOKYO are 1.15% and 5.66%, respectively. This indicates that low-level features may degrade detection performance. From Tables 4 and 5, it is evident that halfway fusion provides the best performance. Comparing halfway fusion with other fusion methods, it can be concluded that halfway fusion effectively extracts and fuses features from both modalities.

#### CFCM module

The CFCM module assists one modality in obtaining complementary feature information from another modality during the feature extraction process. To validate the effectiveness of this module, we conducted an ablation study. The CFCM module was applied during the feature extraction stage of the two sub-networks. The specific deployment is shown in Fig. 1. In this section, the experiments are based on the architecture in Fig. 1, utilizing different numbers of CFCM modules to fuse and supplement feature maps from different modalities.

As shown in Table 4, the more CFCM modules used in the feature extraction network, the lower the MR, and the better the performance of the detection network. The baseline, which does not use CFCM modules, has MR values of 11.23%, 10.84%, and 11.47% for the reasonable, reasonable daytime, and reasonable nighttime subsets, respectively. In the YOLO_CMN architecture, the MR values are 7.85%, 8.03%, and 7.82%, respectively. Compared to the baseline, the miss rates are reduced by 3.38%, 2.81%, and 3.65%, respectively.

**Table 4:** Impact of the number of CFCM modules.

Number of CFCM			MR (reasonable)		
0	1	2	All	Day	Night
✓	✗	✗	11.23	10.84	11.47
✗	✓	✗	9.11	9.52	9.03
✗	✗	✓	7.85	8.03	7.82

**Fig. 12:**
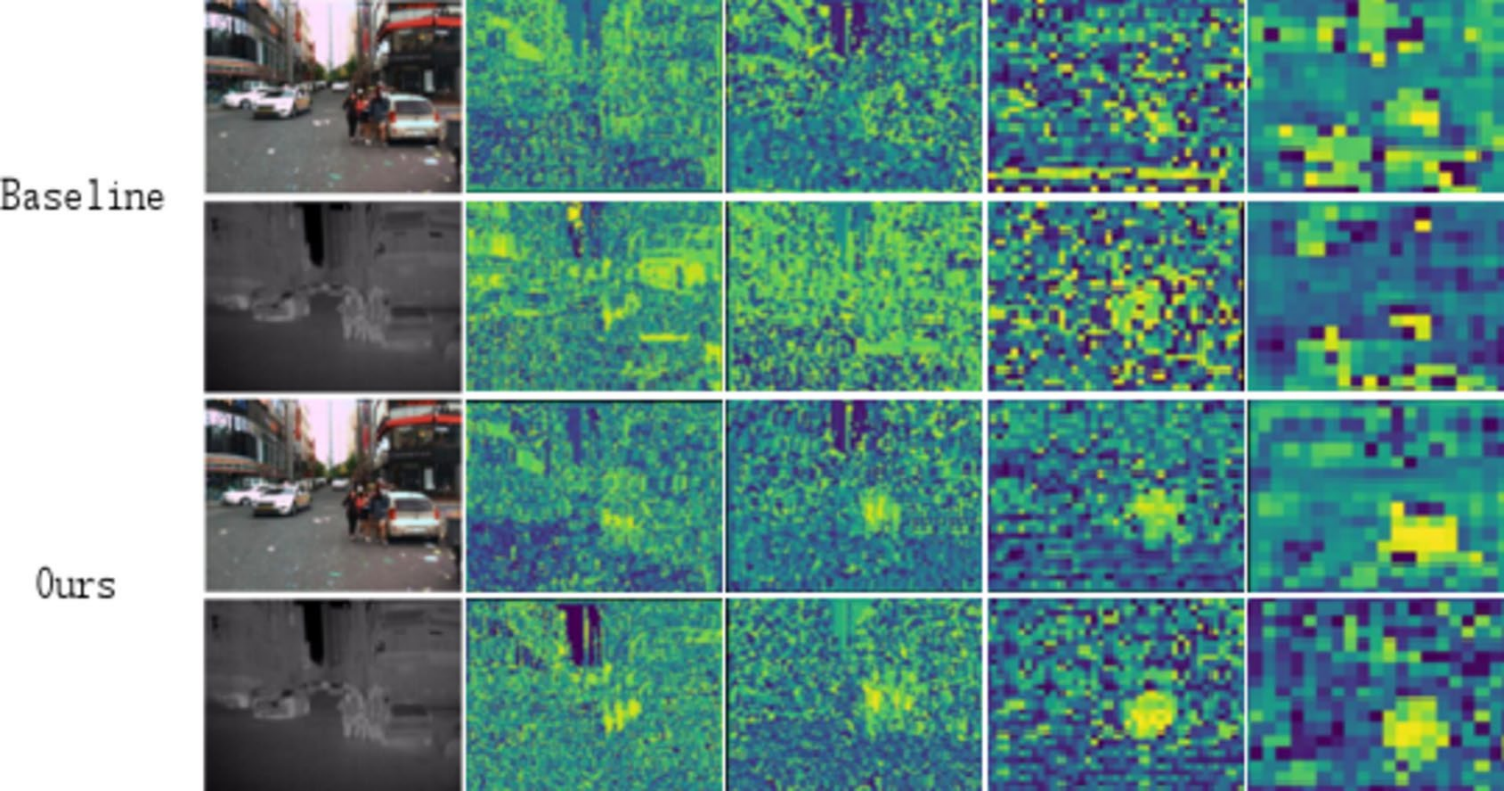
Overall perspective view of the baseline and OURS feature mapping.

After executing the convolutional blocks of the backbone network, feature maps are generated, as shown in Fig. 12. A comparison of the feature maps from the baseline method and the proposed method clearly demonstrates that the inclusion of the CFCM module enhances pedestrian features while suppressing background features. The annotations in Fig. 12 highlight areas where pedestrian features are more distinct and where background noise is effectively suppressed, providing readers with a more intuitive understanding of the role of the CFCM module.

Specifically, under poor lighting conditions in RGB images, extracting pedestrian features becomes particularly challenging, while pedestrian features in thermal images are more prominent. The CFCM module learns thermal image features while extracting pedestrian features from the RGB modality, leveraging the complementary characteristics of both modalities to enable the learning of richer feature information.

These visual comparisons aim to demonstrate how the CFCM module facilitates feature refinement and background noise suppression in deeper network layers. Readers should focus on how the CFCM module preserves and enhances pedestrian features in low-light conditions while suppressing background information. These results indicate that the CFCM module promotes modality interaction within the network, reduces target loss, highlights pedestrian features, minimizes redundant learning, transmits more information, and ultimately improves detection performance under various lighting conditions.

In this paper, the MCSF adaptively selects features from both the color stream and the thermal stream based on lighting conditions to detect pedestrians. To evaluate the effectiveness of the MCSF, we compared it with two other fusion methods, SUM and MIN, based on our proposed semi-fusion architecture. The evaluation of these fusion methods was conducted using both the KAIST and UTOKYO datasets, assessing performance not only under reasonable settings but also under all settings. The comparison of miss rate results is shown in Table 5.

**Table 5:** Miss rate comparison of different fusion methods on KAIST and UTOKYO datasets under reasonable and all settings.

Method	KAIST		UTOKYO	
	Reasonable	All	Reasonable	All
SUM	5.75	21.50	23.69	27.05
MIN	5.22	20.94	23.20	27.26
MCSF	4.91	20.64	23.14	26.21

From the Table 5, it is evident that the MCSF method outperforms both the SUM and MIN fusion methods across both datasets and settings. This demonstrates the superiority of MCSF in adaptively selecting the most relevant features for pedestrian detection based on varying lighting conditions, thus enhancing the overall detection performance.

**Fig. 13:**
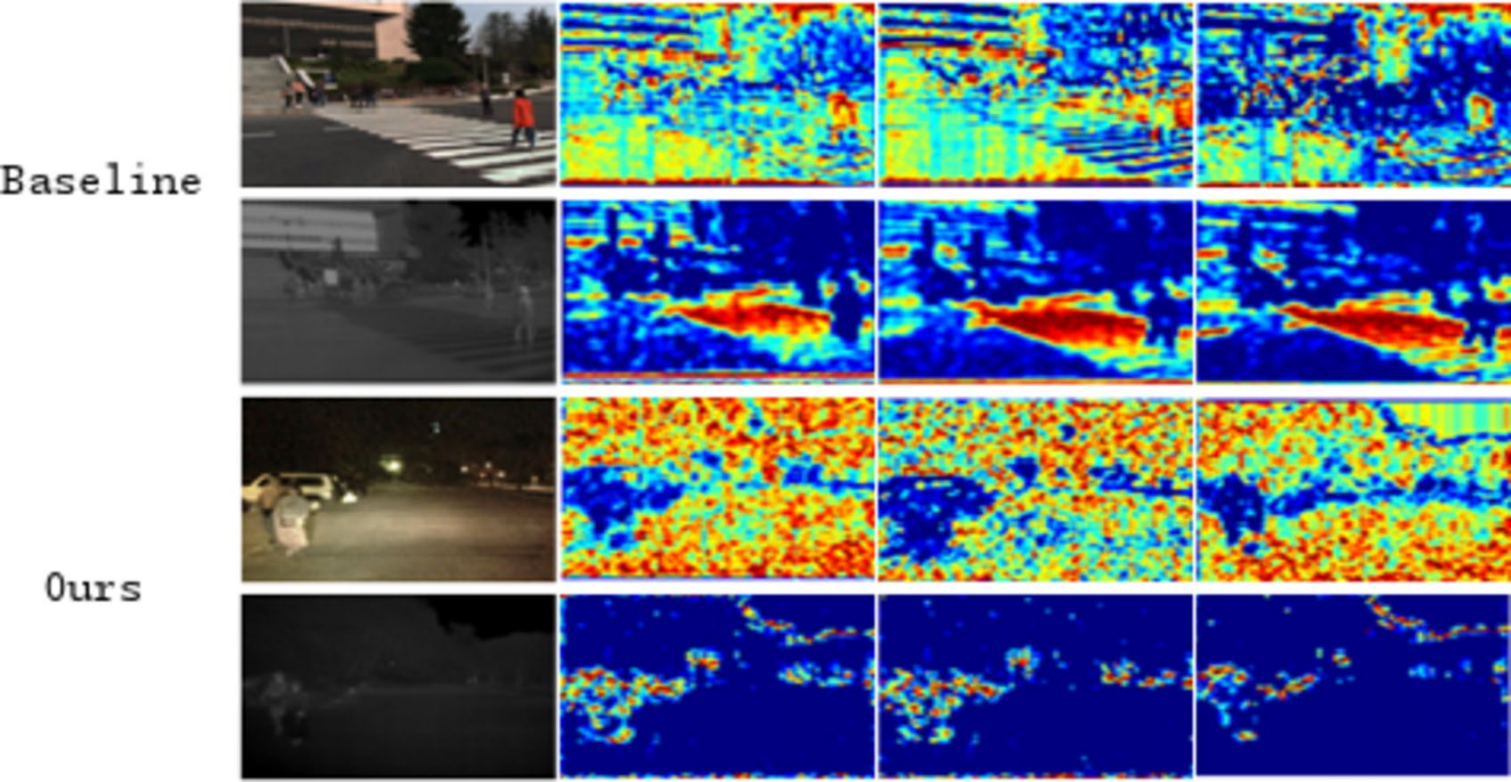
Two modal feature mappings complement each other.

On the KAIST dataset, the proposed method achieves significant performance improvements, with relative gains of 14.6% and 6% compared to other multispectral pedestrian fusion approaches. The feature mappings at Stage 3, shown in Fig. 13, demonstrate that the fused features encapsulate richer semantic information compared to single-modal features, effectively enhancing feature representation.

Similarly, on the UTOKYO dataset, the semi-fusion architecture achieves performance improvements of 3% and 11%, respectively. The MCSF module exhibits consistently superior performance compared to other fusion methods, such as SUM and MIN, across all experimental settings on both datasets, highlighting its robustness and adaptability in integrating multispectral information.

The visualizations in Fig. 13 illustrate the complementary nature of color and thermal modalities. Key regions in the feature maps reveal how the fused features integrate distinct information from both modalities, resulting in improved feature representations. These results validate the effectiveness of the MCSF module in achieving enhanced feature fusion, leading to significant advancements in detection accuracy and robustness under diverse conditions.

These ablation studies demonstrate that the architecture exhibits excellent detection performance and rapid detection speed. Overall, the network strikes a good balance between detection accuracy and speed, making it applicable in practical engineering scenarios.

## Conclusion

This paper proposes a cross-modality detection network for all-weather pedestrian detection. A low-cost CFCM module is added to the feature extraction stage of Yolov7, facilitating information exchange between different modalities during feature extraction. Consequently, the network can achieve complementary information flow between the two modes at the feature extraction stage, reducing target loss. Additionally, the MCSF module is introduced to fuse the color and thermal streams, further enhancing object features and reducing detection errors. The basic features of the two modes are learned through enhancement and suppression processes. By leveraging the complementary features of color and thermal images and multi-scale fusion of deep feature layers, the network achieves multi-dimensional data mining in both horizontal and vertical directions of the parallel deep network. This enriches the depth semantic information of the targets, improving the detector’s performance.

Experimental results demonstrate that the proposed model effectively fuses visible and infrared features and can detect pedestrians of various scales under varying lighting conditions and occlusions. Furthermore, experimental studies on different multispectral fusion strategies have shown that halfway fusion exhibits the best performance in multispectral pedestrian detection. By combining information from infrared sensors and visible light cameras, halfway fusion achieves more accurate and comprehensive pedestrian detection. The results indicate that this method effectively overcomes the limitations of single sensors, enhancing the accuracy and robustness of pedestrian detection. This fusion method leverages the advantages of infrared sensors under low light or nighttime conditions and visible light cameras under daytime or well-lit conditions, enabling all-weather and multi-scenario pedestrian detection. Thus, halfway fusion technology demonstrates excellent performance and broad application prospects in multispectral pedestrian detection.

Future work will include exploring more rational attention mechanisms to more effectively fuse dual-modality features for improved detection performance and developing lighter modules to enhance the network’s detection speed.

## Data Availability

The datasets used and/or analyzed during the current study available from the corresponding author on reasonable request.
